# GABA_A_ Receptor-Mediated Sleep-Promoting Effect of Saaz–Saphir Hops Mixture Containing Xanthohumol and Humulone

**DOI:** 10.3390/molecules26237108

**Published:** 2021-11-24

**Authors:** Byungjick Min, Yejin Ahn, Hyeok-Jun Cho, Woong-Kwon Kwak, Hyung Joo Suh, Kyungae Jo

**Affiliations:** 1 Department of Integrated Biomedical and Life Science, Graduate School, Korea University, Seoul 02841, Korea; bj.min@lotte.net (B.M.); cassandra7@hanmail.net (Y.A.); suh1960@korea.ac.kr (H.J.S.); 2 Lotte R&D Center, Seoul 07594, Korea; hyeokjun_cho@lotte.net (H.-J.C.); wkkwak@lotte.net (W.-K.K.); 3 Transdisciplinary Major in Learning Health Systems, Graduate School, Korea University, Seoul 02841, Korea

**Keywords:** hop, Saaz, Sapir, xanthohumol, humulone, GABA_A_ receptor

## Abstract

Hops contain flavonoids that have sedative and sleep-promoting activities such as α-acid, β-acid, and xanthohumol. In this study, the sleep-enhancing activity of a Saaz–Saphir hops mixture was measured. In the caffeine-induced insomnia model, the administration of a Saaz–Saphir mixture increased the sleep time compared to Saaz or Saphir administration alone, which was attributed to the increase in NREM sleep time by the δ-wave increase. Oral administration of the Saaz–Saphir mixture for 3 weeks increased the γ-amino butyric acid (GABA) content in the brain and increased the expression of the GABA_A_ receptor. As the GABA antagonists picrotoxin and bicuculline showed a decrease in sleep activity, it was confirmed that the GABA_A_ receptor was involved in the Saaz–Saphir mixture activity. In addition, the GABA_A_ receptor antagonist also reduced the sleep activity induced by xanthohumol and humulone contained in the Saaz–Saphir mixture. Therefore, xanthohumol and humulone contained in the Saaz–Saphir mixture showed sleep-promoting activity mediated by the GABA_A_ receptors. The mixture of the Saaz and Saphir hop varieties may thus help mitigate sleep disturbances compared to other hop varieties.

## 1. Introduction

People in modern organizational society deal with excessive stress due to complex interpersonal relationships, heavy workloads, and fatigue. Excessive stress causes psychological instability and disrupts the balance of the autonomic nervous system, which maintains homeostasis, leading to cardiovascular and cerebrovascular diseases, hormonal imbalance, and sleep disturbance [1,2,3]. Sleep is essential for regulating the physiological processes in the human body, and a lack of sleep causes physical problems, such as gastrointestinal diseases, muscle pain, and fatigue, as well as mental problems such as anxiety and depression. Stress can also cause sleep disorders in people [4,5]. There are many causes of sleep disturbances, such as stress, tension, and anxiety. Benzodiazepine drugs such as benzodiazepine receptor agonists and antidepressants with sedative actions are used to treat these sleep disorders. The use of these sleep-disrupting drugs is not suitable for people experiencing early or temporary sleep disturbances, because tolerance and dependence are formed when these drugs are used for a long time [6,7]. Therefore, the development of various natural products that can improve sleep disorders with few side effects has progressed.

Among the natural products showing sleep-promoting activity, hops have been used as nervous stabilizers, hypnotic agents, anti-inflammatory agents, and blood sugar-lowering agents since ancient times [8]. The yellow-green flowers of hops (*Humulus lupulus* L.) belonging to the *Cannabinaceae* family are used as raw materials for beer and give beer its characteristic bitter taste and aroma [9]. It has been reported that the sedative activity of hops is mainly due to flavonoids such as bitter acids and xanthohumol. α-bitter acids are composed of humulone (35–70%), cohumulone (20–65%), and adhumulone (10–15%) and are known to have anti-inflammatory, anticancer, and antibacterial effects, as well as sedative effects [10]. In particular, humulone has been reported to act directly on GABA_A_ receptors [11]. Xanthohumol binds to GABA_A_ receptors and exhibits sleep-promoting activity and is known to exhibit antioxidant and anti-inflammatory effects [12,13]. Differences occur in the polyphenol content, the major component, of hops and depend on the harvest time and variety. Therefore, different hop varieties show different sleep-promoting activities, depending on the flavonoid content.

We selected hop varieties Saaz and Saphir based on previous studies, which reported that these varieties have excellent sleep-promoting activities among industrially used hop varieties. Among the selected hops mixtures, the administration of a 75:25 mixture of Saaz–Saphir showed high sleep-promoting activity in the pentobarbital-induced sleep test. In this study, the effect of the Saaz–Saphir mixture (75:25) on sleep time and quality was elucidated by a EEG analysis in the rodent model, and the receptors involved in sleep activity were elucidated through an antagonist-binding analysis.

## 2. Results

### 2.1. Sleep Latency and Sleep Duration of the Saaz–Saphir Mixture

Post the hop active ingredient content analysis, the content of xanthohumol was slightly higher in the Saphir extract than in the Saaz extract, and isoxanthohumol was not detected in the hop extracts (Table 1 and Figure S1). Except for cohumulone in the components corresponding to α-acids and β-acids, the contents of the Saphir extract were higher than that of Saaz. Among them, the content of total α-acid was 70.60 mg/g of the extract in Saaz and 83.94 mg/g of the dry extract in Saphir (Figures S2 and S3). Since the Saaz–Saphir mixture was in the ratio of 75:25, the active substances of Saphir were lower in concentration compared to that of the Saaz extract in the mixture.

**Figure 1:**
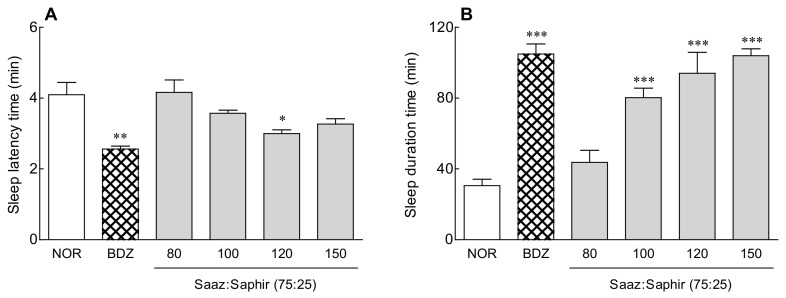
Effects of hops on the sleep latency time (**A**) and total sleep time (**B**) in mice that received a hypnotic dose of pentobarbital (42 mg/kg, i.p.). NOR: the 0.9% NaCl (physiological saline) group (normal control); BDZ: benzodiazepine treatment (positive control, 200 μg/kg); and Saaz–Saphir mixtures (80, 100, 120, and 150 mg/kg). Values are presented as the mean ± standard errors of the mean (SEM) for each group, *n* = 7. * *p* < 0.05, ** *p* < 0.01, and *** *p* < 0.001 vs. NOR by Tukey’s multiple comparison test.

The most effective Saaz–Saphir mixture (75:25) was selected for a sleep enhancement activity evaluation (Figure S4). The sleep latency time and sleep duration were measured by oral administration of 80–150 mg/kg of Saaz–Saphir mixture (75:25), respectively, to sleep-induced mice by pentobarbital (Figure 1). The sleep latency time showed a tendency to increase as the concentration of the Saaz–Saphir mixture administration increased, and it was significantly lower than that of the saline oral administration group (NOR) (*p* < 0.05) at 120-mg/kg administration.

The sleep duration showed a tendency to increase as the Saaz–Saphir mixture concentration increased, and the sleep duration increased significantly higher than that of the NOR group (*p* < 0.001) when more than 100 mg/kg of the mixture was administered. Administration of the Saaz–Saphir mixture at 120 and 150 mg/kg was effective in reducing the sleep latency time and increasing the sleep duration compared to oral administration at other concentrations.

### 2.2. EEG Analysis by Administration of the Saaz–Saphir Mixture

**Figure 2:**
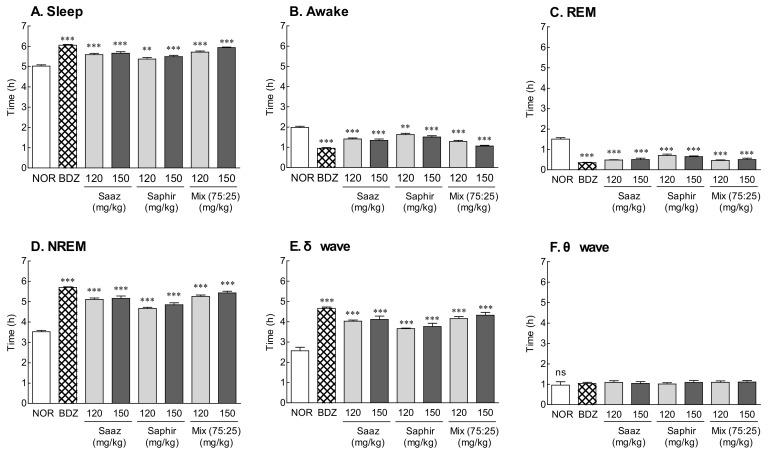
Effects of hops (Saaz, Saphir, and Saaz–Saphir mixture) on the electrophysiologic pattern in rats. Electroencephalography (EEG) analyses were conducted for 9 days. (**A**) Sleep time, (**B**) awake time, (**C**) duration of REM, (**D**) duration of NREM, (**E**) δ wave of NREM, and (**F**) θ wave of NREM. NOR: the 0.9% NaCl (physiological saline) group (normal group), BDZ: benzodiazepine treatment (positive control, 200 μg/kg), Saaz (120 and 150 mg/kg), Saphir (120 and 150 mg/kg), and the Saaz–Saphir mixture (75:25, 120 and 150 mg/kg). Values are presented as the mean ± standard error of the mean (SEM) for each group, *n* = 8. Asterisks indicate significant differences: ** *p* < 0.01, and *** *p* < 0.001 vs. NOR by Tukey’s multiple comparison test. NS, not significant.

The EEG was analyzed after the oral administration of 120 and 150 mg/kg of the Saaz–Saphir mixture to measure the total sleep time, REM, and NREM sleep time (Figure 2). The oral administration of the Saaz–Saphir mixture significantly increased the sleep time and significantly decreased the waking time compared to that of the NOR group (*p* < 0.001). The increase in sleep time on the Saaz–Saphir mixture administration was attributed to the increase in NREM sleep time according to the increase in δ waves. The administration of 150 mg/kg of the mixture was more effective in promoting sleep than the administration of 120 mg/kg of the mixture. The oral administration of 150 mg/kg of Saaz–Saphir showed a tendency to increase the sleep time compared to an oral administration of 150 mg/kg of Saaz or Saphir alone, and the δ waves contributing to NREM also tended to increase.

### 2.3. EEG Analysis by Administration of the Saaz–Saphir Mixture in the Caffeine-Induced Insomnia Model

**Figure 3:**
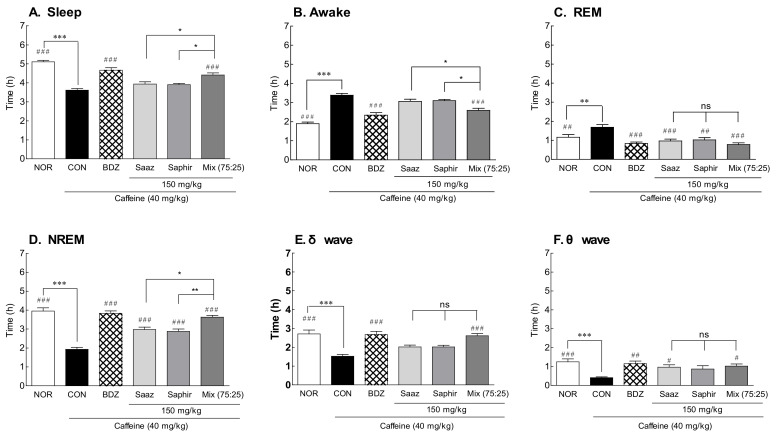
Effects of hops (Saaz, Saphir, and Saaz–Saphir mixture) on electrophysiologic patterns in rat-induced insomnia by caffeine. Electroencephalography (EEG) analyses were conducted for 4 days (**A**) Sleep time, (**B**) awake time, (**C**) duration of REM, (**D**) duration of NREM, (**E**) δ wave of NREM, and (**F**) θ wave of NREM. NOR: the 0.9% NaCl (physiological saline) group (normal control), CON: caffeine control (40 mg/kg), BDZ: Benzodiazepine treatment (positive control, 200 μg/kg), Saaz (120 and 150 mg/kg), Saphir (120 and 150 mg/kg), and Saaz–Saphir mixture (75:25, 120 and 150 mg/kg). Values are presented as the mean ± standard error of the mean (SEM) for each group, *n* = 8. Asterisks indicate significant differences: *** *p* < 0.001 vs. NOR; * *p* < 0.05 and ** *p* < 0.001 vs. Saaz–Saphir mixture; and ^#^ *p* < 0.05, ^##^ *p* < 0.01, and ^###^ *p* < 0.001 vs. CON by Tukey’s multiple comparison test.

An EEG analysis was performed by administering the Saaz–Saphir mixture to insomnia-induced rats with caffeine (Figure 3). As there was a significant difference in the sleep time and waking time between the NOR group and CON (a group with saline administered to insomnia-induced rats), it was confirmed that the insomnia model was induced by caffeine (Figure 3A; *p* < 0.001). The awake time tended to decrease in the insomnia model caused by caffeine when Saaz or Saphir alone was administered orally, and the sleep time tended to increase, but there was no significant difference compared with the CON group. However, when Saaz or Saphir was administered alone, the REM sleep time significantly decreased (*p* < 0.001 and *p* < 0.01, respectively), and the NREM sleep time significantly increased (*p* < 0.001). There was a significant increase in sleep time compared to the CON group (*p* < 0.001) when the Saaz–Saphir mixture was administered. In addition, there was a significant difference in the sleep times between the groups administered Saaz or Saphir alone (*p* < 0.05). The administration of hop extracts showed a tendency to increase the sleep time due to the increases in NREM sleep compared to that of the CON group. In particular, the increase in NREM sleep in the hop mixture-administered groups compared with that in CON group was attributed to the significant increase in δ and θ waves. According to the above results, administration of the Saaz–Saphir mixture showed better sleep-enhancing effects than the administration of Saaz or Saphir alone.

### 2.4. Changes of GABA and Receptor Expression Levels in Mouse Brain by Oral Administration of the Saaz–Saphir Mixture

**Figure 4:**
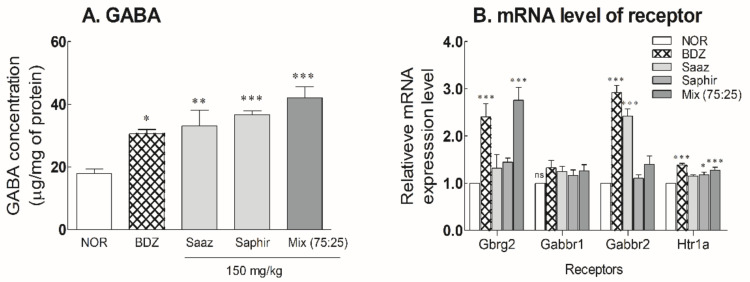
Effects of hops extracts on the content of (**A**) GABA and (**B**) mRNA expressions of GABAergic and serotonergic receptors in mice. NOR: the 0.9% NaCl (physiological saline) group (normal control), BDZ: Benzodiazepine treatment (positive control, 200 μg/kg), Saaz (150 mg/kg), Saphir (150 mg/kg), and Saaz–Saphir (75:25, 150 mg/kg). Values are presented as the mean ± standard error of the mean (SEM) for each group, *n* = 6. Asterisks indicate significant differences: * *p* < 0.05, ** *p* < 0.01, and *** *p* < 0.001 vs. NOR by Tukey’s multiple comparison test. NOR: Normal, BDZ: Benzodiazepine, Gabrg2: GABA_A_ receptor subunit gamma 2, Gabbr1: GABA_B_ receptor subunit 1, Gabbr2: GABA_B_ receptor subunit 2, and Htr1a: 5-hydroxytryptamine receptor 1A.

After oral administration of the 150-mg/kg Saaz–Saphir mixture for 3 weeks, the changes in the GABA content and receptor expression levels in the mouse brain were measured (Figure 4 and Figure S5). The GABA content in the mouse brain significantly increased due to the administration of hop extract alone or the hop mixture (*p* < 0.01 and *p* < 0.001, respectively) compared to the NOR group. The administration of hop extracts exhibited a higher level of GABA content in the mouse brain than when BDZ 200 μg/kg was administered. The GABA level in the mouse brain tended to be higher during the Saaz–Saphir mixture administration than that of Saaz or Saphir administration alone (Figure 4A).

The expression levels of receptors following the oral administration of hops extracts were significantly higher than those of the NOR group when the Saaz–Saphir mixture was administered (Figure 4B). The increased expression of GABA_A_ and 5-HT1A receptors by oral administration of the Saaz–Saphir mixture indicates the potential of the mixture to bind to these receptors.

### 2.5. Receptor Binding Mode of the Saaz–Saphir Mixture by Antagonists

**Figure 5:**
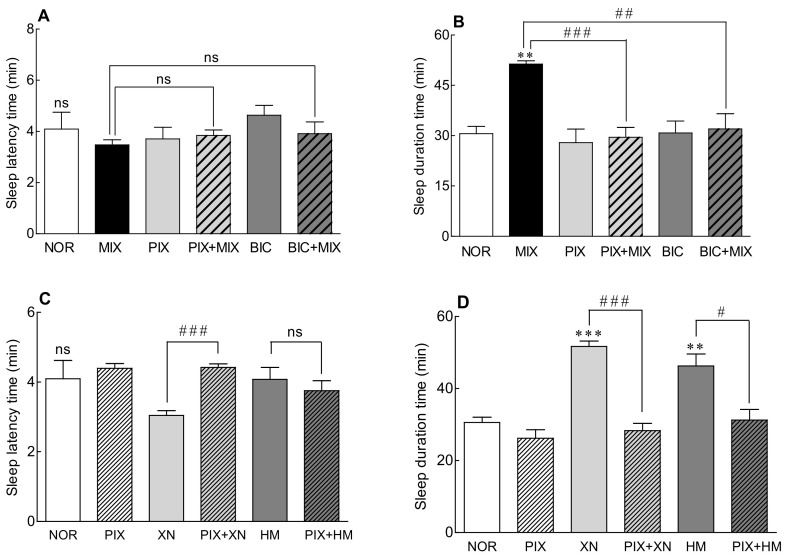
Effects of the GABA_A_ receptor antagonist on the sleep latency time (**A**,**C**) and total sleep time (**B**,**D**) in mice that received a hypnotic dose of pentobarbital (42 mg/kg, i.p.). NOR: the 0.9% NaCl (physiological saline) group (normal control), MIX (Saaz:Saphir = 75:25, 150 mg/kg), PIX (4 mg/kg), and BIC (6 mg/kg). Values are presented as the mean ± standard error of the mean (SEM) for each group, *n* = 7. Asterisks indicate significant differences: ** *p* < 0.01 and *** *p* < 0.001 vs. NOR and ^#^ *p* < 0.05, ^##^ *p* < 0.01, and ^###^ *p* < 0.001 between the antagonist treatment and non-antagonist treatment (MIX group) by Tukey’s multiple comparison test. NOR, normal; MIX, mixture; PIX, picrotoxin; BIC, bicuculline; XN, xanthohumol; HM, humulone; and ns, not significant.

As the expression level of the GABA_A_ receptor increased when the Saaz–Saphir mixture was administered (Figure 4B), the change in the sleep-promoting activity of the Saaz–Saphir mixture was measured by the addition of picrotoxin and bicuculline, antagonists of the GABA_A_ receptor (Figure 5). When picrotoxin or bicuculline was administered alone, there was no significant difference in the sleep latency time and sleep duration compared to that of the NOR group, so it was confirmed that the antagonists did not participate in sleep promotion. The Saaz–Saphir mixture administration showed no significant difference in the sleep latency time, but the sleep duration showed a significant difference with that of the NOR group (*p* < 0.01). The Saaz–Saphir mixture administration, which increased the sleep duration time, exhibited significantly decreased sleep duration when Saaz–Saphir was administered with picrotoxin or bicuculline (*p* < 0.001 and *p* < 0.01, respectively). Therefore, the sleep enhancing activity of the mixture seemed to be mediated by the GABA_A_ receptor, as the antagonists picrotoxin and bicuculline reduced the sleep duration time of the Saaz–Saphir mixture.

Alcoholic extracts of Saaz and Saphir, which constitute the Saaz–Saphir blend, contain the active substances xanthohumol (2.51 and 4.23 mg/g, respectively) and humulone (47.23 and 53.13 mg/g, respectively; Table 1). Changes in the sleep activity were measured when picrotoxin was added to hop active substances such as xanthohumol and humulone, respectively (Figure 5C,D). When xanthohumol or humulone was administered alone, the sleep duration was significantly longer than that of the NOR group (*p* < 0.001 and *p* < 0.01, respectively). However, when picrotoxin was added to xanthohumol and humulone, the sleep duration time was significantly reduced compared to xanthohumol or humulone administration alone (*p* < 0.001 and *p* < 0.05, respectively). According to the above results, it is estimated that the Saaz–Saphir mixture containing xanthohumol and humulone exhibits sleep-promoting activity mediated by the picrotoxin-binding site among the GABA_A_ receptors.

## 3. Discussion

Hop cones are female flowers that are rich in resins, essential oils, and polyphenols and contribute to the distinctive aroma and bitter taste of beer. Hop resins contain various active ingredients, which are divided into hard and soft resins according to solvent solubility.

Hard resins contain a large amount of xanthohumol, the main component of hops, and soft resins are divided into α-bitter acids (humulone, cohumulone, and adhumulone) and β-bitter acids (lupulone, colupuline, and adlupulone). These ingredients differ depending on the hop variety, and their physiological activities are also different. In this study, the sleep-promoting activity of 10 hop extracts, including Saaz and Saphir, was measured in a fruit fly model, and Saaz, Saphir, and Simcoe were selected. A Saaz–Saphir mixture (75:25) was selected by measuring the sleep latency time and sleep duration in a pentobarbital-induced sleep model for a mixture of the three selected hops. The sleep-promoting activity and mechanism of action of the Saaz–Saphir mixture (75:25) was thus studied.

Saaz–Saphir mixed administration showed significant differences in sleep latency and sleep duration changes in the 120- and 150-mg/kg treatment groups compared to the normal group (Figure 1). The EEG analysis in the normal model also showed an increase in the sleep time and NREM sleep time compared to the single administration of the components constituting the mixture, but there was no significant difference in these times (Figure 2). However, administration of the Saaz–Saphir mixture in the caffeine-induced insomnia model showed differences in the sleep time and awake time compared to Saaz or Saphir administration alone (Figure 3). The Saaz–Saphir mixture was more effective in improving the sleep disorders than Saaz or Saphir administration alone, as shown in Figure 4, with an increase in the sleep time and NREM time in the insomnia model.

Caffeine is a widely used stimulant [14]. Caffeine is a methlxanthine-based compound that increases arousal, induces cortical activation, and reduces fatigue. Caffeine induces arousal by blocking the action of adenosine receptors in the basal forebrain and causes a decrease in the slow-wave activity of sleep in the cortex [15]. The CON group showed a decrease in delta and theta waves corresponding to slow waves, and slow-wave sleep was recovered by administration of the Saaz–Saphir mixture.

A 30% decrease in the occipital lobe GABA concentration was reported in people with sleep disturbances compared to normal people [16,17]. Additionally, a decrease in the ratio of GABA to creatine in the frontal lobe of shift workers has been reported compared to day workers [18]. The anterior hypothalamus and the anterior visual region are involved in the initiation of sleep, and sleep-promoting substances include GABA, adenosine, acetylcholine, and serotonin. The ventrolateral preoptic nucleus of the anterior hypothalamus contains GABA as a neurotransmitter, which is activated during sleep and inhibits monoamine and histamine secretion regions to prevent waking. The thalamic reticular nucleus also contains GABA as a neurotransmitter and generates sleep spindles [19]. Therefore, GABA is closely related to sleep. Administration of the Saaz–Saphir mixture showed an increase in the GABA content in the rat brain (Figure 4A), which contributes to the sleep-enhancing effect.

After the oral administration of Saaz–Saphir for 3 weeks, an increase in the expression level of the GABA_A_ receptor was confirmed. On the other hand, Htr1a, a serotonin receptor, interacts with serotonin as a ligand, a derivative of tryptophan [20]. Htr1a was upregulated by the Saaz–Saphir treatment at the mRNA level but showed a relatively insignificant level compared to the GABA_A_ receptor (Figure 4B). Based on these results, picrotoxin and bicuculline, which are antagonists of the GABA_A_ receptor, showed changes in the sleep duration when administered together with the Saaz–Saphir mixture, which appears to exhibit sleep-promoting activity owing to the involvement of the GABA_A_ receptor (Figure 5).

GABA receptors can be divided into GABA_A_, GABA_B_, and GABA_C_ receptors, and the physiological actions of GABA appear mostly through GABA_A_ receptors. GABA_A_ receptors play an important role in sedation, sleep, and anesthesia [21,22,23]. The GABA_A_ receptor is a highly complex ligand-gated ion channel and consists of five subunits that can belong to eight different subunit classes known as α1-6, β1-4, γ1-3, ρ1-3, δ, ε, π, and φ, and combinations of different subunits exhibit different functions [24]. The actions of GABA_A_ receptors are regulated by allosteric modulators such as barbiturate, benzodiazepine, neurosteroids, bicuculline, picrotoxin, and zinc. Picrotoxin is a non-transferential antagonist of the GABA_A_ receptor, which directly antagonizes the GABA_A_ receptor channel, a ligand-gated ion channel involved in the passage of chloride ions through cell membranes. It promotes inhibitory effects on target neurons by preventing Cl-channel permeability. Bicuculline is a competitive antagonist of the GABA_A_ receptor, which antagonizes the binding of GABA to the GABA_A_ receptor [25]. Bicuculline also blocks calcium-activated potassium channels [26]. Therefore, in this study, it was evaluated whether Saaz–Saphir was blocked by GABA_A_ receptor antagonists picrotoxin and bicuculline. According to the findings, the hops mixture may show sleep-promoting activity mediated by GABA_A_ receptors, as the sleep duration was changed by the Saaz–Saphir mixture administration, along with picrotoxin or bicuculline administration.

Most sedative hypnotics used to treat insomnia target the GABA_A_ receptor [27]. However, pharmacological treatments can cause undesirable side effects such as addiction, tolerance, and dependence. Therefore, with the aim of developing safe sleeping pills, research on hypnotic drugs based on the current GABA_A_ receptors and positive effects of natural products on the treatment of sleep disorders have been reported [28]. It is known that xanthohumol contained in hops enhances sleep by increasing the GABA activity through the regulation of GABA_A_ receptors [12]. Humulone, another active ingredient in hops, has sedative/hypnotic effects and acts as a positive allosteric modulator of the GABA_A_ receptors. Xanthohumol and humulone therefore act on the GABA_A_ receptors to exhibit sleep-promoting activity. As sleep activity was reduced by picrotoxin, a GABA_A_ receptor antagonist, it is estimated that xanthohumol and humulone, which are sleep-active substances contained in hops, exhibit sleep-promoting activity by binding to the picrotoxin site of the GABA_A_ receptor (Figure 5C,D). However, although the content of xanthohumol in the Saaz–Saphir mixture is lower than that in Saphir, the sleep-promoting activity of the Saaz–Saphir mixture was high, so it is presumed that components other than xanthohumol are also involved in sleep. Additional studies should be conducted to identify the active substances other than xanthohumol.

The above results indicate that the Saaz–Saphir mixture had a better sleep promoting effect than Saaz or Saphir administration alone in the caffeine-induced model corresponding to the sleep disturbance model. Xanthohumol, the main component of the hop mixture, appears to show sleep-promoting activity by binding to the picrotoxin site of the GABA_A_ receptor. Therefore, the Saaz–Saphir mixture, the raw material for brewing beer, may help improve sleep disorders.

## 4. Materials and Methods

### 4.1. Preparation of Hops Alcoholic Extract

The hop pellet samples Saaz and Saphir were provided by the Lotte R&D Center (Seoul, Korea). Hops dried at 50–60 °C (moisture content 8–10%) were crushed using a hammer mill and then pelletized. Ethanol (70%), corresponding to 25 times the amount of hops (200 g), was added, followed by a reflux extraction at 95 °C for 1 h, and the reflux extraction was performed twice. The extract was then filtered (Whatman No. 1), concentrated under reduced pressure, and lyophilized.

### 4.2. Flavonoids Assay Using HPLC

Xanthohumol and isoxanthohumol were measured according to a previously described method [29]. The mobile phases used were (A) 0.025% trifluoroacetic acid in water and (B) 0.025% trifluoroacetic acid in acetonitrile. The mobile phase was passed through a YMC-Triart C18 column (4.6 × 150 mm, 5 μm) at a flow rate of 1 mL/min. The sample injection volume was 5 μL, and detection was done at a wavelength of 372 nm. The samples were injected at a concentration of 2 mg/mL.

The α-Acids and β-acids were analyzed as previously described [30]. For the analysis, a YMC-Triart C18 column (150 × 4.6 mm, 5 μm) (Wilmington, NC, USA) was used; the column temperature was 35 °C, and the injection volume was 20 μL. The samples were injected at a concentration of 2.5 mg/mL. The mobile phase (methanol:water:phosphoric acid = 775/210/9, *v/v/v*) was passed for 45 min at a flow rate of 1.0 min/mL, and the detection wavelength was 275 nm. The humulone standard was obtained from AOBIOUS (AOBIOUS INC, Gloucester, MA, USA), and the international calibration extract ICE3 was purchased from Labor Veritas Co. (Zürich, Switzerland). ICE3 contained 44.64% of the total α-acid (cohumulone: 13.68% and adhumulone: 30.76%) and 24.28% of the total β-acid (colupuone: 13.44% and adlupulone: 10.84%).

### 4.3. Pentobarbital-Induced Sleep Test in Mouse

The Institute of Cancer Research (ICR) male mice (6-week-old) were purchased from Orient Bio (Sungnam, Korea). In the animal breeding room, the temperature was 21 ± 1 °C, the relative humidity was 50–55%, a 12-h cycle of light and dark was maintained, and drinking water and food were provided ad libitum. The animals were used for the experiments after a one-week adaptation period. After fasting for 24 h before the pentobarbital-induced sleep test, the hops extract was orally administered 40 min before the pentobarbital injection, and pentobarbital was injected intraperitoneally at a concentration of 42 mg/kg to measure the sleep latency and sleep duration [29]. The sleep time was recorded until recovery of the upright reflex, and the experimental animals whose sleep was not induced within 15 min after pentobarbital administration were excluded from the experiment. All animal experiments were conducted with the approval of the Korea University Institutional Animal Care and Use Committee (KUIACUC-2021-0020).

### 4.4. Electroencephalogram (EEG) Analysis

The EEG analysis was performed according to a previously described experimental method [14]. Sprague–Dawley (SD) male rats (5-week-old) purchased from Orient Bio were anesthetized by inhalation with isoflurane, and EEG electrodes were inserted. After the operation was completed, the animals were allowed to recover for a week, and then, an EEG transmitter (EMKA Technologies, Paris, France) was attached. The EEG was measured at 15 mm/s for 7 h from 10 a.m. to 5 p.m. from the time the sample was orally administered.

The EEG was also analyzed in the caffeine-induced SD rat arousal model. The experimental groups were a normal control group (NOR), a negative control group (CON, caffeine-treated group), a positive control group (BDZ), and a group administered with the Saaz–Saphir mixture (Mix 75:25). Caffeine at a concentration of 10 mg/kg was administered together with the hops sample in all the groups except for the NOR group [29]. At this time, alprazolam, a benzodiazepine of 0.2 mg/kg, was administered to the BDZ group.

### 4.5. Analysis of the Receptor Binding Mode Using an Antagonist

An experiment on the receptor binding mode by the antagonist was carried out in a pentobarbital-induced sleep model. The normal control group (NOR) was treated with saline, and the Saaz–Saphir mixture (75:25) was administered at 150 mg/kg and xanthohumol (Sigma-Aldrich, St Louis, MO, USA) and humulone (Aobious, Gloucester, MA, USA) at 20 mg/kg. Using edible oil, 6 mg/kg of bicuculline (Sigma-Aldrich) and 4 mg/kg of picrotoxin (Sigma-Aldrich) were intraperitoneally injected (i.p.) 30 min before hop extract administration [31]. Sleep latency and sleep duration were measured under the same conditions as in the pentobarbital sleep induction experiment.

### 4.6. Receptor Gene Expression Level and GABA Content Analysis

Male ICR mice (25 g, 6-week-old) in the BDZ group were orally administered 0.2 mg/kg and 150 mg/kg of each hop extract (Saaz, Saphir, and hops mixture (Saaz:Saphir = 75:25)) for 3 weeks and sacrificed to measure the receptor expression and GABA levels.

Total RNA was extracted from the brain tissue using the TRIzol^®^ reagent (Invitrogen, Carlsbad, CA, USA), and cDNA was synthesized using the Reverse Transcription System (Promega, Madison, WI, USA). The synthesized cDNA was subjected to real-time PCR using the TaqMan^®^ Universal PCR Master Mix (Applied Biosystems, Foster, CA, USA) and a real-time PCR system (7500, Applied Biosystems, Foster, CA, USA). The real-time PCR TaqMan probes used were as follows: GABA_A_ receptor subunit gamma 2 (Gabrg2, NM_008073.3), GABA_B_ receptor 1 (Gabbr1, NM_019439.3), GABA_C_ receptor subunit rho 2 (Gabbr2, NM_008076.3), 5-hydroxytryptamine receptor 1A (Htr1a, NM_008308.4), and GAPDH (NM_001289726.1).

The GABA content in the brain was determined by a HPLC analysis using a Waters AccQ-Tag column (3.9 mm × 150 mm), a Waters 2475 Multi λ Fluorescence Detector (Milford, MA, USA) (250-nm excitation and 395-nm emission wavelengths), and a Waters AccQ-Tag system (Waters Corp., Milford, MA, USA). Mobile phases A, B, and C were water AccQ-tag eluent A (acetate–phosphate buffer), acetonitrile, and Milli-Q water, respectively [32].

### 4.7. Statistical Analysis

All results were expressed as the mean ± standard mean error (SEM), and significance was verified post hoc by Tukey’s new multiple range test using IBM SPSS 16.0 (Chicago, IL, USA).

## Figures and Tables

**Table 1:** Xanthohumol, isoxanthohumol, α-acid, and β-acid contents (mg/g of dry extract) of the Saaz and Saphir ethanol extracts.

Hop	Xanthohumol	α-Acids			β-Acids	
		Humulone	Cohumulone	Adhumulone	Colupulone	Adlupulone
Saaz	2.51 ± 0.13	47.28 ± 0.23	5.41 ± 0.01	17.91 ± 0.05	20.68 ± 0.05	28.83 ± 0.53
Saphir	4.23 ± 0.14	53.13 ± 0.20	3.14 ± 0.09	27.67 ± 0.24	27.12 ± 0.07	39.24 ± 0.10

Values are presented as the mean ± standard deviation (SD).

## Data Availability

The data that support the findings of this study are available from the corresponding author upon reasonable request.

## Supplementary Materials

The following are available online. Figure S1: HPLC chromatograms of (A) standard xanthohumol, (B) Saaz ethanol extract, and (C) Saphir ethanol extract. Figure S2: HPLC chromatograms of (A) standard humulone, (B) Saaz ethanol extract, and (C) Saphir ethanol extract. Figure S3: HPLC chromatograms of (A) standard, (B) Saaz ethanol extract, and (C) Saphir ethanol extract. 1: cohumulone; 2: adhumulone; 3: colupulone; 4: adlupulone. Figure S4: Effects of hops on sleep latency time (A) and total sleep time (B) in mice that received a hypnotic dose of pentobarbital (42 mg/kg, i.p.). NOR: 0.9% NaCl (physiological saline) group (normal control), BDZ: benzodiazepine treatment (positive control, 200 μg/kg), Saaz 100 mg/kg, Saphir 100 mg/kg, Saaz-Saphir mixture (75:25, 50:50, and 25:75; 100 mg/kg). Values are presented as the mean ± standard error of the mean (SEM) for each group, *n* = 7. ** *p* < 0.01 and *** *p* < 0.001 vs. NOR by Tukey’s multiple comparison test. Figure S5: HPLC chromatograms of (A) standard GABA, (B) Saaz extract treated group, (C) Saphir extract treated group, and (D) Saaz-Saphir mixture treated group.

## Sample Availability

Samples of the ethanolic hop extract are available from the first author, Byungjick Min.
