# Supplementary material for: GABAA Receptor-Mediated Sleep-Promoting Effect of Saaz–Saphir Hops Mixture Containing Xanthohumol and Humulone
Source: Molecules. 2021 Nov 24;26(23):7108. doi: 10.3390/molecules26237108 (PMC8659287; doi:10.3390/molecules26237108)
Supplement: Supplementary file 1 [file molecules-26-07108-s001.zip › molecules-1441291-supplementary.pdf]

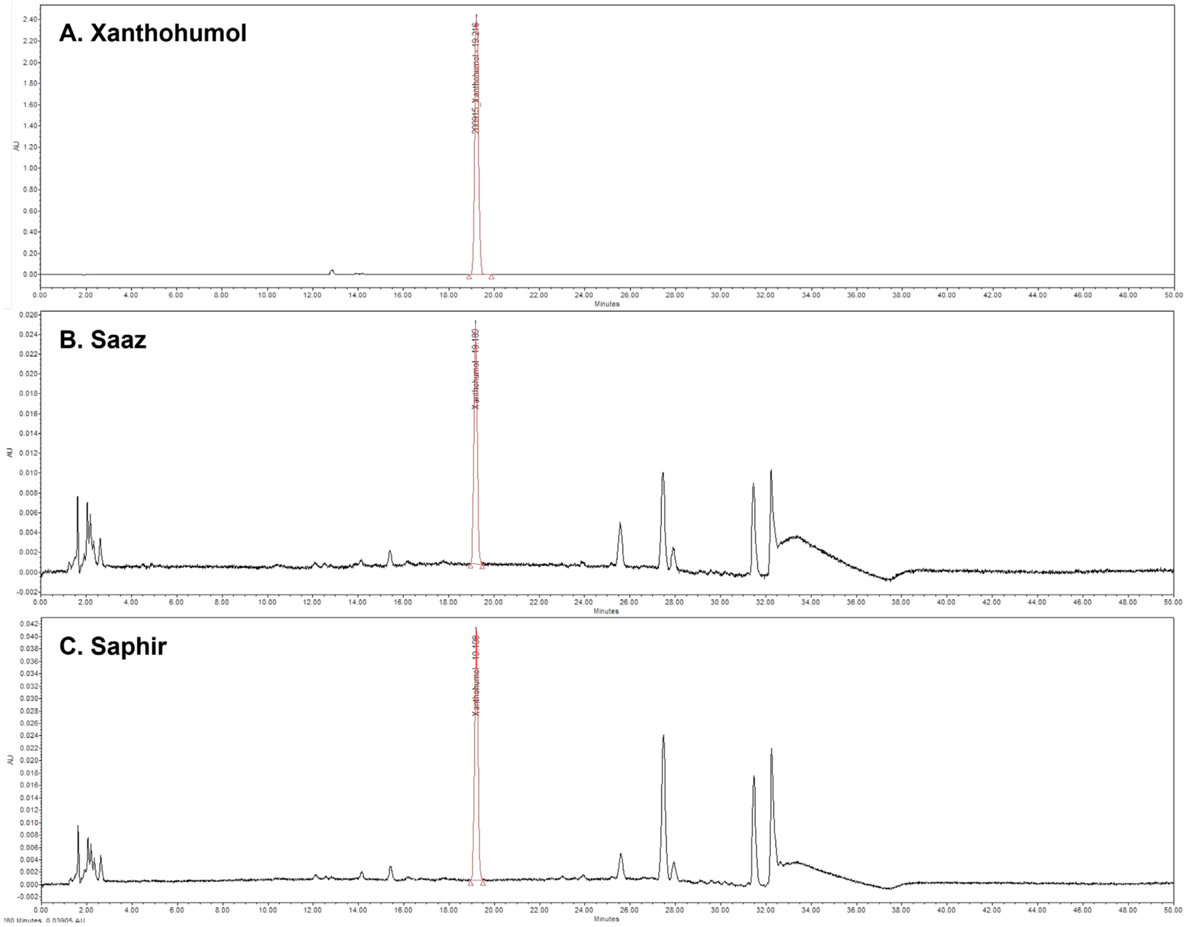

Figure S1: HPLC chromatograms of (A) standard xanthohumol, (B) Saaz ethanol extract, and (C) Saphir ethanol extract.

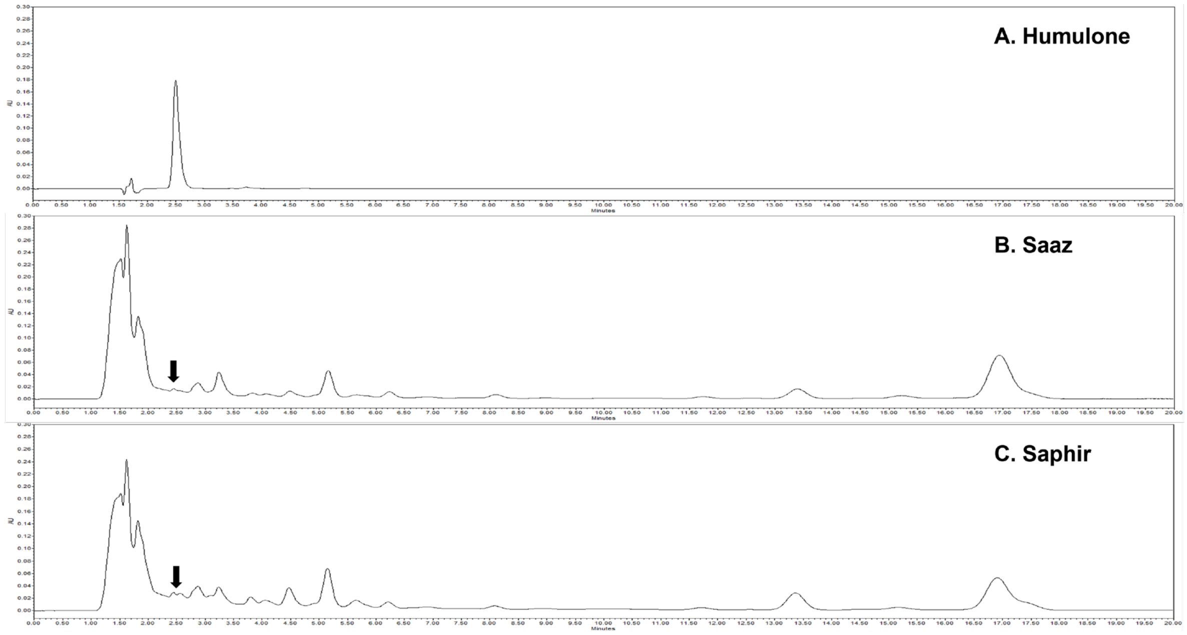

Figure S2: HPLC chromatograms of (A) standard humulone, (B) Saaz ethanol extract, and (C) Saphir ethanol extract.

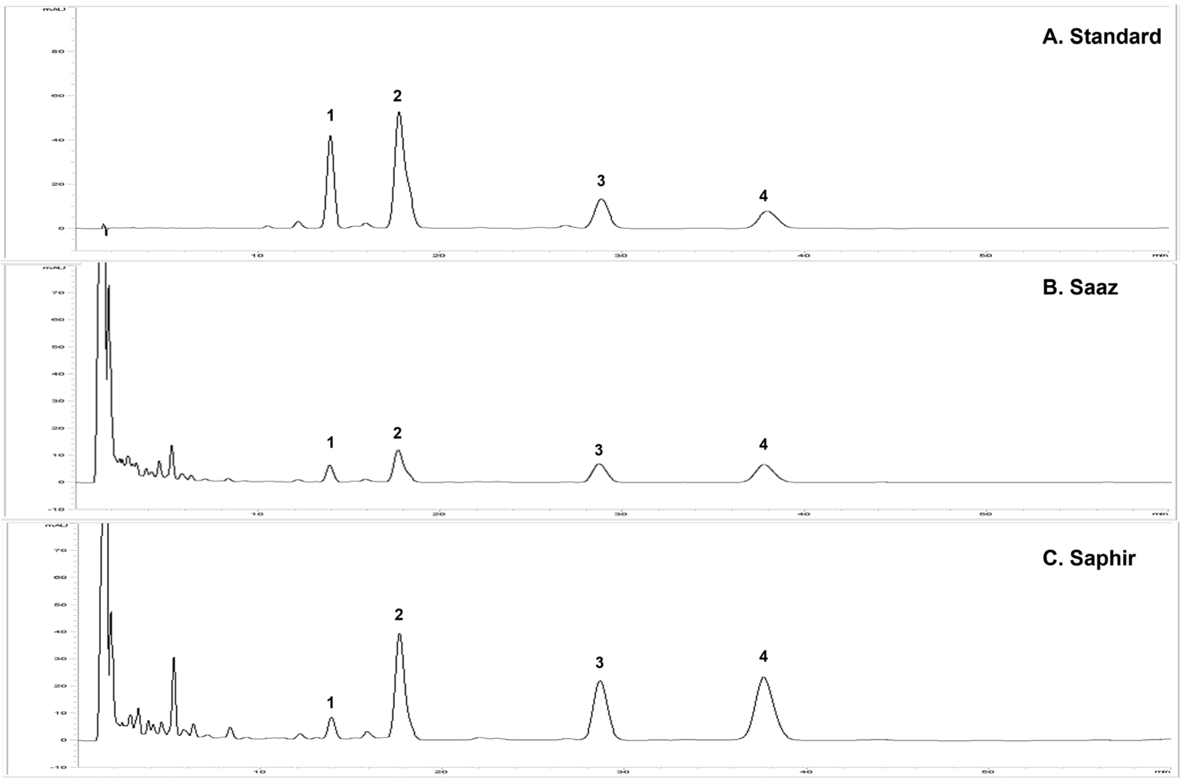

Figure S3: HPLC chromatograms of (A) standard, (B) Saaz ethanol extract, and (C) Saphir ethanol extract. 1: cohumulone; 2: adhumulone; 3: colupulone; 4: adlupulone.

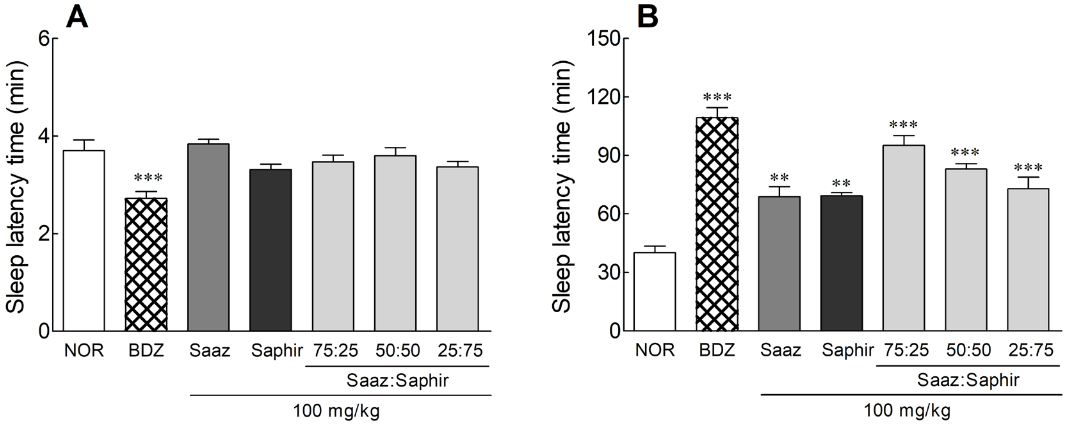

Figure S4: Effects of hops on sleep latency time (A) and total sleep time (B) in mice that received a hypnotic dose of pentobarbital (42 mg/kg, i.p.). NOR: 0.9% NaCl (physiological saline) group (normal control), BDZ: benzodiazepine treatment (positive control, 200 μg/kg), Saaz 100 mg/kg, Saphir 100 mg/kg, Saaz-Saphir mixture (75:25, 50:50, and 25:75; 100 mg/kg). Values are presented as the mean ± standard error of the mean (SEM) for each group, n=7. **p < 0.01 and ***p < 0.001 vs. NOR by Tukey's multiple comparison test.

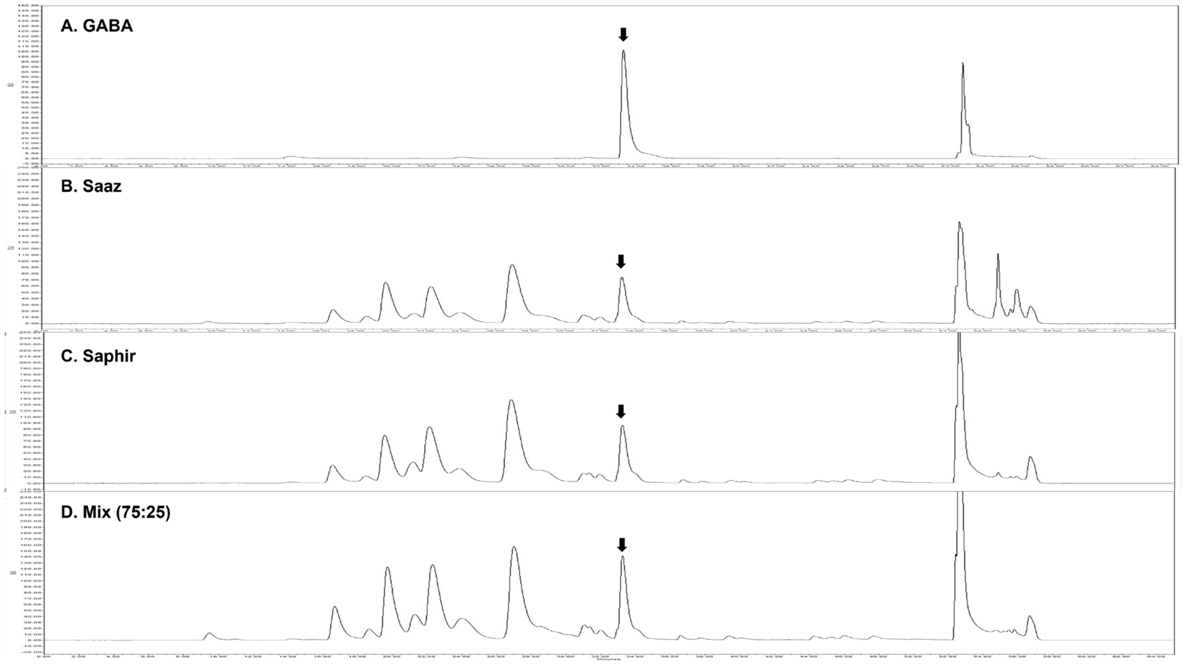

Figure S5: HPLC chromatograms of (A) standard GABA, (B) Saaz extract treated group, (C) Saphir extract treated group, and (D) Saaz-Saphir mixture treated group.
